# Busulfan and thiotepa as a conditioning regimen for autologous stem cell transplantation in patients with multiple myeloma: A study of the Korean Multiple Myeloma Working Party (KMMWP-1801 study)

**DOI:** 10.3389/fonc.2022.959949

**Published:** 2022-08-30

**Authors:** Ga-Young Song, Sung-Hoon Jung, Jin Seok Kim, Hyeon Seok Eom, Joon Ho Moon, Ho-Young Yhim, Kihyun Kim, Chang-Ki Min, Je-Jung Lee

**Affiliations:** ^1^ Department of Hematology-Oncology Chonnam National University Hwasun Hospital and Chonnam National University Medical School, Hwasun, South Korea; ^2^ Severance Hospital, Yonsei University College of Medicine, Seoul, South Korea; ^3^ National Cancer Center, Goyang-si, South Korea; ^4^ Kyungpook National University Hospital, School of Medicine, Kyungpook National University, Daegu, South Korea; ^5^ Division of Hematology/Oncology Chonbuk National University Medical School, Jeonju, South Korea; ^6^ Samsung Medical Center, Sungkyunkwan University School of Medicine, Seoul, South Korea; ^7^ Seoul St. Mary’s Hematology Hospital, Catholic University of Korea, Seoul, South Korea

**Keywords:** multiple myeloma, autologous stem cell transplantation, conditioning regimen, busulfan and melphalan, melphalan conditioning

## Abstract

**Background:**

Autologous stem cell transplantation (ASCT) remains the standard of care for patients with newly diagnosed multiple myeloma (MM). Several attempts to improve the efficacy of conditioning regimens have been conducted in MM, but no more effective regimen than conventional high-dose melphalan has been introduced.

**Objective:**

In this study, the efficacy and toxicity of busulfan and thiotepa (BuTT) and those of high-dose melphalan (HD-MEL) were compared retrospectively as a conditioning regimen for ASCT in patients with MM.

**Study design:**

Included in the analysis were 114 patients who received BuTT and 114 patients who received HD-MEL treatment between March 2008 and May 2020. The BuTT regimen consisted of intravenous thiotepa 5 mg/kg once a day from days 7 to 6, followed by intravenous busulfan 3.2 mg/kg once a day from days 5 to 3. The HD-MEL conditioning regimen consisted of melphalan 100 mg/m^2^ once a day from days 3 to 2.

**Results:**

The overall response rate after ASCT did not differ between BuTT and HD-MEL (94.7% in BuTT vs. 97.4% in HD-MEL, *p* = 0.333). After a median follow-up of 47.6 months, progression-free survival (PFS) tended to be longer in the BuTT group (median PFS, 41.5 months vs. 30.3 months; hazard ratio (HR), 0.706; 95% confidence interval (CI), 0.497–1.004, *p* = 0.053). In the subgroup analysis of patients who did not proceed to maintenance or consolidation treatment after ASCT, the difference in PFS became more significant (median PFS, 41.5 months vs. 24.4 months; HR, 0.621; 95% CI, 0.388–0.993; *p* = 0.047). Additionally, the BuTT group had fewer adverse events, such as grade 3 or 4 stomatitis and diarrhea, than the HD-MEL group (stomatitis, 10.5% vs. 23.7%, *p* = 0.013; diarrhea, 10.5% vs. 25.4%, *p* = 0.005). There was no difference in the occurrence of venous-occlusive disease (2.6% in BuTT vs. 0.9% in HD-MEL, *p* = 0.622).

**Conclusion:**

Our study results suggest that BuTT is an effective alternative conditioning regimen with reduced toxicity in patients with newly diagnosed MM.

## Introduction

Multiple myeloma (MM), in which monoclonal plasma cells proliferate in the bone marrow, producing an overabundance of monoclonal paraprotein, is a hematologic malignancy that has seen significant therapeutic advances over the past 20 years (1). Although the advent of novel therapies such as immunomodulatory agents, proteasome inhibitors, and monoclonal antibodies has made it possible to obtain a deeper therapeutic response in newly diagnosed MM patients, autologous stem cell transplantation (ASCT) produces an additive effect to achieve an enhanced response.

However, in contrast to the numerous advances in novel therapeutic agents, the conditioning regimen used in ASCT has remained relatively unchanged. Currently, the standard conditioning regimen for ASCT in MM is high-dose melphalan (HD-MEL; melphalan 200 mg/m^2^). To date, no specific conditioning regimen superior to HD-MEL has been introduced. Melphalan dosage is usually reduced to 100 mg/m^2^ or 140 mg/m^2^ in elderly or physically inactive patients due to toxicity conditions, such as mucositis. However, it is unclear whether the reduced dose of melphalan is as effective as a conventional dose of melphalan (200 mg/m^2^) (2–5). The number of transplant-eligible patients has increased dramatically, and more older patients are now receiving ASCT due to the advances in supportive care and the development of novel agents. As a result, a new conditioning regimen that is both effective and less toxic is required.

A thiotepa-containing regimen of thiotepa, busulfan, and cyclophosphamide (TBC) has demonstrated efficacy in treating lymphoid malignancies (6–8). The TBC regimen has also been studied in ASCT for MM patients. However, despite its effectiveness, it is not used frequently due to toxicity complications (9–11). Double alkylator-based regimens in which more agents are added to melphalan, such as busulfan and melphalan (BUMEL) or thiotepa and melphalan, appear to be safe and effective. However, due to melphalan supply constraints, busulfan and thiotepa (BuTT) conditioning as another combination of alkylating agents was commonly used in South Korea.

Here, the outcome of ASCT after BuTT and HD-MEL conditioning were compared over different periods in MM patients.

## Materials and methods

### Patients and treatment

Between 2007 and 2021, 114 patients underwent ASCT receiving BuTT conditioning regimens from seven institutions in Korea. For comparison, the same number of patients who received ASCT with the HDMEL conditioning regimen between 2007 and 2021 was retrospectively recruited from the same institutions. Patients with systemic light chain (AL) amyloidosis or plasma cell leukemia were excluded from the analysis. Patients with no measurable disease, defined as serum M-protein ≥0.5 g/dl or urine M-protein ≥200 mg/24 h before induction treatment were excluded. Patients who received tandem ASCT or allogeneic stem cell transplantation were also excluded. The BuTT conditioning regimen consisted of intravenous thiotepa 5 mg/kg once a day from days 7 to 6, followed by intravenous busulfan 3.2 mg/kg once a day from days 5 to 3. The HD-MEL conditioning regimen was comprised of melphalan 100 mg/m^2^ once a day from days 3 to 2. Response to treatment was assessed after induction chemotherapy before ASCT and 3 months after ASCT. Maintenance therapy was administered based on the policies of each participating center. This study was approved by the institutional review board of each participating institution and was conducted in accordance with the Declaration of Helsinki.

### Definitions

The Revised International Staging System (R-ISS) was used to assess the clinical disease stage at diagnosis (12). Cytogenetic risk at diagnosis was classified into high-risk and standard-risk based on conventional cytogenetic studies or fluorescent *in situ* hybridization. Patients with del(17p), t (4,14), t (14,16), or amp(1q) were defined as having high-risk chromosomal abnormalities. A response assessment was conducted according to the International Myeloma Working Group uniform response criteria (13). Response assessment was made after induction treatment and on day 100 after transplantation before starting consolidation or maintenance treatment. The overall response rate (ORR) was defined as the proportion of patients who achieved more than a partial response (PR). Time to neutrophil engraftment was defined as the duration between the date of transplantation and the first day of three consecutive days in which the absolute neutrophil count exceeded 0.5 × 10^9^/L without the administration of a granulocyte-colony stimulating factor. Time to platelet engraftment was defined as the duration between the date of transplantation and the first day of seven consecutive days in which the platelet count was >20 × 10^9^/L without platelet transfusion. Adverse events were graded according to the National Cancer Institute Common Terminology Criteria for Adverse Events (v5.0). Venous-occlusive disease (VOD) was diagnosed using both the Seattle and Baltimore criteria, and the severity was assessed using modified Seattle criteria (14, 15).

### Statistical analysis

Discrete and continuous variables were evaluated using Fisher’s exact test and the Mann–Whitney *U*-test. Progression-free survival (PFS) was defined as the period from the date of transplantation to the date of disease progression, or death from any cause. Overall survival (OS) was defined as the period from the date of transplantation to the date of the last follow-up or death from any cause. PFS and OS were investigated using the Kaplan–Meier method and compared using the log-rank test. The two patients who received BuTT in the second ASCT were excluded from the survival analysis. The relative risk of an event and its 95% confidence interval (CI) were estimated using the Cox proportional hazard model. Variables of *p <*0.1 in univariate analyses were included in the multivariate analysis. A *P*-value <0.05 was considered statistically significant in all analyses. All of the statistical computations were performed using SPSS software (ver. 21; SPSS Inc., Chicago, IL, USA).

## Results

### Patients

One hundred and fourteen patients received BuTT conditioning, and the same number of patients received HD-MEL. The baseline clinical characteristics of patients did not differ significantly between the two groups (**Table 1**). The number of patients with high-risk chromosomal abnormalities came to 35 (32.4%) in the BuTT group and 31 (28.2%) in the HD-MEL group (*p* = 1.000). The proportion of the patients with extramedullary plasmacytoma at diagnosis was higher in the BuTT group (24.6% vs. 14.9%, *p* = 0.095). Because the clinical outcomes of the BuTT group were compared with those of the historical control group who received HD-MEL induction treatment before ASCT, there was a difference between the two groups. All of the BuTT patients received a bortezomib-containing induction regimen of bortezomib, thalidomide, and dexamethasone (VTD); bortezomib, cyclophosphamide, and dexamethasone (VCD); and bortezomib and dexamethasone (VD) (VTD, 86.0%; VCD, 5.2%; VD, 8.8%). As for the HD-MEL group, 75 patients (65.8%) received bortezomib-containing treatment of VTD, VCD, VD, or bortezomib, melphalan, and prednisone (VMP) (VTD, 43.9%; VCD, 18.4%; VD, 0.9%; VMP, 2.6%), and the other patients received a thalidomide-based treatment of thalidomide, cyclophosphamide, and dexamethasone (TCD); thalidomide and dexamethasone (TD); or only cytotoxic chemotherapy such as vincristine and doxorubicin, dexamethasone (VAD) (TCD or TD, 28.9%; VAD, 5.3%). None of the patients in the BuTT group received a reduced-dose conditioning regimen, and four patients (3.5%) in the HD-MEL group needed melphalan dose reduction (140 mg/m^2^) because of poor general condition (one patient) and renal impairment (three patients). More patients in the HD-MEL group received more than one line of treatment prior to ASCT (13.2% vs. 1.8%, p = 0.002), but all of the patients who received two prior treatments before ASCT in the HD-MEL group did not respond to the first thalidomide-based treatment and changed to a bortezomib-based regimen. More patients in the HD-MEL group received thalidomide maintenance after ASCT than in the BuTT group (50.0% vs. 28.1%, *p* = 0.001).

**Table 1:** Baseline clinical characteristics according to conditioning regimen.

Variables	BuTT(n = 114)	HD-MEL(n = 114)	*p*-value
Median age, year (range)	56 (34–64)	55 (27–67)	0.706
Male, n (%)	62 (54.4)	62 (54.4)	1.000
Immunoglobulin (Ig) type, n (%)			0.735
IgG	61 (53.5)	69 (60.5)	
IgA	18 (15.8)	16 (14.0)	
IgD	5 (4.4)	5 (4.4)	
Light chain only	30 (26.3)	24 (21.1)	
R-ISS, n (%)			0.657
I	31 (28.2)	29 (25.4)	
II	58 (52.7)	67 (58.8)	
III	21 (19.1)	18 (15.8)	
Cytogenetics, n (%) High-risk Standard-risk	35 (32.4) 73 (67.6)	31 (28.2) 79 (71.8)	1.000
ECOG PS ≥2, n (%)	4 (3.5)	10 (8.5)	0.166
LDH > (1 × ULN), n (%)	22 (19.8)	30 (26.3)	0.271
Median BM plasma cells, %, range	35.5 (1.0–99.0)	40.0 (2.6–96.3)	0.590
β2-microglobulin, mg/L	3.8 (1.4–41.9)	3.5 (1.1–27.7)	0.514
Plasmacytoma, n (%)	28 (24.6)	17 (14.9)	0.095
Serum creatinine ≥2 mg/dl, n (%)	12 (10.6)	14 (12.3)	0.835
Median platelet count (×10^9^/L), range	208 (44–701)	196 (40–460)	0.624
Hb <10 g/dl, n (%)	60 (53.1)	64 (56.1)	0.690
Median time to transplant, months (range)	6.0 (4.1–41.2)	6.2 (3.2–10.0)	0.833
Mobilization regimen, n (%)			<0.001
G-CSF	45 (39.5)	29 (25.4)	
G-CSF + ETO	62 (54.4)	19 (16.7)	
G-CSF + CY	6 (5.3)	61 (53.5)	
Plerixafor	1 (0.9)	5 (4.4)	
Year of transplantation			<0.001
2007–2011	0 (0.0)	33 (28.9)	
2012–2016	37 (32.5)	32 (28.1)	
2017–2021	77 (67.5)	49 (43.0)	
Conditioning regimen dose reduction, N (%)	0 (0.0%)	4 (3.5%)	–
Prior line of therapy before transplantation, n (%)			0.002
1	112 (98.2)	99 (86.8)	
2	2 (1.8)	15 (13.2)	
Bortezomib before transplantation, n (%)	114 (100.0)	75 (65.8)	<0.001
Maintenance treatment after transplantation, n (%)	32 (28.1)	57 (50.0)	0.001
Consolidation treatment after transplantation, n (%)	9 (7.9)	6 (5.3)	0.297

N, number; R-ISS, revised international staging system; ECOG, Eastern Cooperative Oncology Group; PS, performance status; LDH, lactate dehydrogenase; ULN, the upper limit of normal value; BM, bone marrow; Hb, hemoglobin; ASCT, autologous stem cell transplantation; ETO, etoposide; CY, cyclophosphamide.

### Treatment response and survival outcome

**Figure 1:**
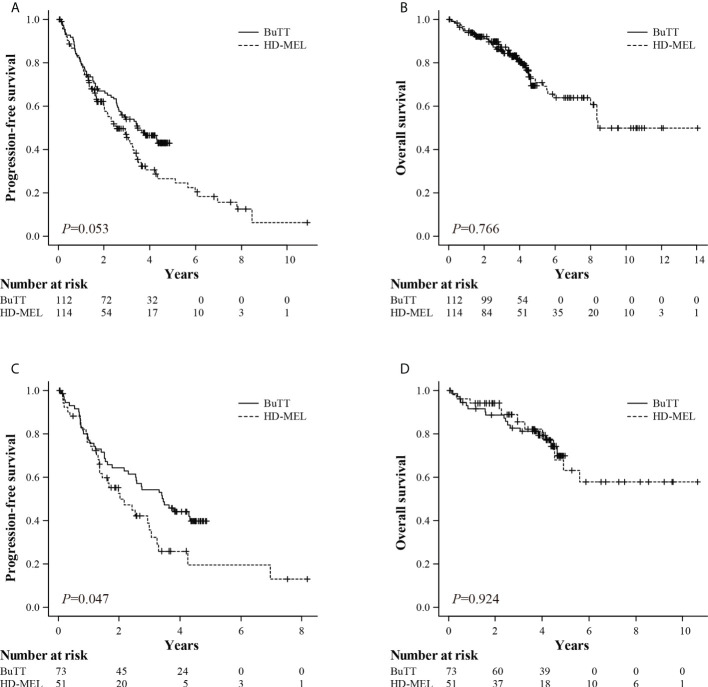
Kaplan–Meier survival curves for progression-free survival (PFS) **(A)** and overall survival (OS) **(B)** for the busulfan and thiotepa (BuTT) group and the high-dose melphalan (HD-MEL) group; PFS **(C)** and OS **(D)** in BuTT and HD-MEL group patients who did not receive maintenance or consolidation treatment after autologous stem cell transplantation (ASCT).

**Figure 2:**
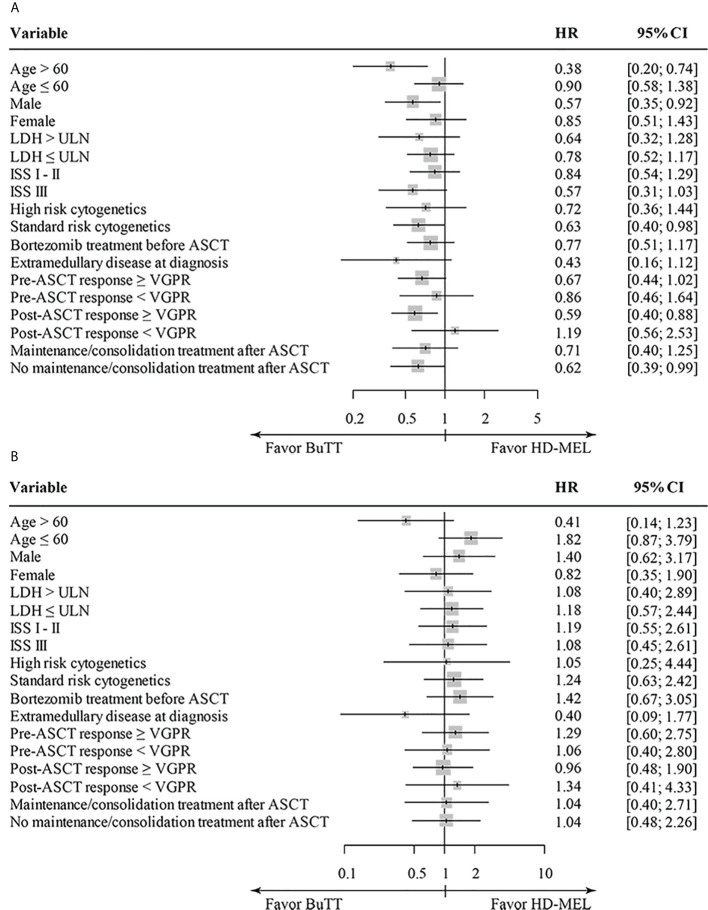
PFS **(A)** and OS **(B)** according to the conditioning regimen of various subgroups. The patients in a subgroup of extramedullary disease at diagnosis included only the patients treated with bortezomib induction therapy before ASCT.

The treatment responses before and after ASCT are described in **Table 2**. The ORR at day 100 after ASCT was 94.7% in the BuTT group and 97.4% in the HD-MEL group (*p* = 0.333). The proportion of patients who achieved more than a very good partial response (VGPR) after ASCT was 79.5% for the BuTT group and 84.2% for the HD-MEL group (*p* = 0.391). Then we analyzed survival outcomes according to the conditioning regimen. For the two patients in the BuTT group, ASCT with BuTT conditioning was their second ASCT. Thus, two patients were excluded from the survival analysis because the clinical outcome after the second ASCT differed from that of the upfront ASCT. After a median follow-up of 47.6 months, the median PFS was 41.5 months in the BuTT group and 30.3 months in the HD-MEL group (hazard ratio (HR) 0.706; 95% CI, 0.497–1.004, *p* = 0.053, **Figure 1A**). There was no difference in OS between the two groups (not reached in the BuTT group vs. 101.0 months in the HD-MEL group; HR, 1.092; 95% CI, 0.610–1.956, *p* = 0.766, **Figure 1B**). In the analysis that included patients who did not proceed to maintenance or consolidation treatment after ASCT, the PFS difference became more significant (41.5 months for the BuTT group vs. 24.4 months for the HD-MEL group; HR, 0.621; 95% CI, 0.388–0.993, *p* = 0.047, **Figure 1C**). The OS did not differ between the two groups (not reached in the BuTT group vs. not reached in the HD-MEL group; HR, 1.038; 95% CI, 0.478–2.255; *p* = 0.924, **Figure 1D**). Because there were more patients in the BuTT group who received bortezomib-containing induction treatment than in the HDMEL group, survival analyses were conducted in the subgroup of patients who were treated with a bortezomib-containing induction regimen. PFS was longer in the BuTT group but the difference was not statistically significant (41.1 months for the BuTT group vs. 35.2 months in the HDMEL group, *p* = 0.246). An improvement in PFS was observed consistently in patients who received BuTT, regardless of age, sex, ISS stage, LDH level at diagnosis, cytogenetic risk group, pre- and post-ASCT response, or maintenance/consolidation treatment (**Figure 2**). Thirty-four patients (29.8%) in the BuTT group and 41 patients (36.0%) in the HD-MEL group were aged more than 60, and the improvement in PFS appeared predominantly in older patients aged more than 60 years. In elderly patients older than 60 years, OS was also better in the BuTT group than in the HD-MEL group. The BuTT group also showed a longer PFS in a subgroup that included patients who had achieved more than VGPR after ASCT (median PFS, 41.8 months vs. 26.1 months; HR, 0.590; 95% CI, 0.400–0.880). There were more patients with extramedullary plasmacytoma at diagnosis in the BuTT group; we analyzed the survival outcomes in patients with extramedullary plasmacytoma who were treated with bortezomib. The BuTT group had a longer PFS than the HD-MEL group in patients with extramedullary plasmacytoma (51.87 months vs. 11.4 months; HR, 0.430; 95% CI, 0.160–1.120).

**Table 2:** Comparison of pre- and post-transplantation responses.

	BuTT (n = 114)		HD-MEL (n = 114)	
	pre	post	pre	post
CR	42.1%	62.9%	33.3%	71.0%
VGPR	34.2%	15.9%	36.8%	13.2%
PR	22.8%	15.0%	21.9%	13.2%
SD	0.9%	4.4%	7.0%	2.6%
PD	0.0%	0.9%	0.9%	0.0%
≥VGPR	76.3%	78.8%	70.1%	84.2%
ORR (≥PR)	99.1%	93.8%	92.0%	97.4%

CR, complete response; VGPR, very good partial response; PR, partial response; SD, stable disease; PD, progressive disease; ORR, overall response rate.

### Engraftment and toxicity

There was no significant difference in hematopoietic stem cell engraftment in either group. The median time to neutrophil engraftment was 10 days (1–28 days) in the BuTT group and 10 days (8–19 days) in the HD-MEL group (p = 0.06). The median time to platelet engraftment was 9 days (1–41 days) in the BuTT group and 9 days (1–40 days) in the HD-MEL group (*p* = 0.403). The post-transplantation hospitalization period was similar in both groups (18 days vs. 19 days, *p* = 0.711). Regarding non-hematologic toxicities, grade 3 or 4 stomatitis and diarrhea occurred more frequently in the HD-MEL group than in the BuTT group (stomatitis, 10.5% vs. 23.7%, *p* = 0.013; diarrhea, 10.5% vs. 25.4%, *p* = 0.005). Grade 3 or 4 of aspartate aminotransferase/alanine aminotransferase (AST/ALT) elevation occurred more frequently in the BuTT group (10.5% vs. 1.8%, *p* = 0.010); however, all of the patients recovered with supportive care. The incidence of infection was higher in the HD-MEL group than in the BuTT group, although there was no statistical difference (50.0% vs. 39.5%, *p* = 0.143). There was no difference in the occurrence of VOD. One patient in each group died within 100 days after ASCT due to pneumonia. The engraftment and toxicity profiles are described in **Table 3**.

**Table 3:** Engraftment and transplant-related non-hematopoietic toxicities.

	BuTT (n = 114)	HD-MEL (n = 114)	*p*-value
Median time to neutrophil engraftment, days (range)	10 (1–28)	10 (8–19)	0.060
Median time to platelet engraftment, days (range)	9 (1–41)	9 (1–40)	0.403
Post-transplantation hospitalization, days (range)	18 (14–62)	19 (13–68)	0.711
Infection, n (%)	45 (39.5)	57 (50.0)	0.143
Gr 1–2 Gr 3–4	27 (23.7) 18 (15.9)	35 (30.7) 22 (19.3)	0.297 0.482
Stomatitis, n (%)	78 (68.4)	93 (81.6)	0.032
Gr 1–2 Gr 3–4	66 (57.9) 12 (10.5)	66 (57.9) 27 (23.7)	1.000 0.013
Nausea, n (%) Gr 1–2 Gr 3–4	96 (84.2) 81 (71.1) 15 (13.2)	97 (85.1) 74 (64.9) 23 (20.2)	1.000 0.394 0.213
Vomit, n (%) Gr 1–2 Gr 3–4	58 (50.9) 55 (48.2) 3 (2.6)	63 (55.3) 58 (50.9) 5 (4.4)	0.596 0.791 0.722
Diarrhea, n (%)	86 (75.4)	97 (85.1)	0.095
Gr 1–2 Gr 3–4	74 (64.9) 12 (10.5)	68 (59.6) 29 (25.4)	0.495 0.005
Constipation, n (%) Gr 1–2 Gr 3–4	20 (17.5) 20 (17.5) 0 (0.0)	14 (12.3) 14 (12.3) 0 (0.0)	0.353 0.353 –
AST/ALT, n (%)	40 (35.1)	37 (32.5)	0.780
Gr 1–2 Gr 3–4	28 (24.6) 12 (10.5)	35 (30.7) 2 (1.8)	0.374 0.010
Bilirubin, n (%)	19 (16.7)	20 (17.5)	1.000
Gr 1–2 Gr 3–4	0 (0.0) 19 (16.7)	0 (0.0) 20 (17.5)	– 1.000
VOD, n (%)	3 (2.6)	1 (0.9)	0.622
Mild Moderate	1 (0.9) 2 (1.8)	0 (0.0) 1 (0.9)	1.000 1.000
Transplantation related mortality, n (%)	1 (0.9)	1 (0.9)	1.000

AST, aspartate aminotransferase; ALT, alanine aminotransferase; VOD, venous-occlusive disease.

## Discussion

This study compared the clinical outcomes of 114 patients who received BuTT as a conditioning regimen for ASCT to those of the same number of patients who received an HD-MEL regimen. The results of this study revealed similar ORR and complete response rates between the two groups. Despite the similar response rate after ASCT, PFS was longer with BuTT conditioning than with HD-MEL, although the difference did not reach statistical significance. Additionally, in subgroup analyses for the patients who did not receive maintenance or consolidation treatment after ASCT or in patients who achieved VGPR after ASCT, the PFS period was significantly longer in the BuTT group. Although there were more patients with plasmacytoma in the BuTT group, the BuTT group consistently showed prolonged PFS in the subgroup analysis for patients with plasmacytoma who had received bortezomib-containing induction treatment. It is difficult to determine whether BuTT is more effective against plasmacytoma due to the small number of patients, and the reason for the improved PFS in patients with plasmacytoma is unclear. Although a large, prospective study is needed to confirm the results, this study is the first to show BuTT to be effective as a conditioning regimen in MM.

HD-MEL is the standard preparative regimen in ASCT for MM. In addition to its established efficacy for MM, HD-MEL requires only one or two days, and a short conditioning schedule has some merits, such as short hospitalization duration, outpatient-based ASCT, and the possibility of using non-frozen stem cells. However, melphalan has its own disadvantages, such as a high incidence of mucositis and the need for dose adjustment for patients with renal impairment (16, 17). Thiotepa is a trifunctional alkylating agent with a broad spectrum of antitumor activity; it has been used mainly as a preparative regimen before ASCT due to its severe bone marrow toxicity (18–20). Thiotepa is known to be active against myeloma when administered at high doses. A thiotepa-containing high-dose treatment regimen, mainly with busulfan and cyclophosphamide, has been examined in earlier studies. The TBC regimen, first introduced in 1993, was effective at extending remission in 53% of MM patients (10). However, in a retrospective study comparing TBC and HD-MEL conducted in 2003, the TBC regimen did not improve clinical outcomes compared with HD-MEL, and the treatment-related mortality rate was higher in the group receiving TBC (6% vs. 1%, *p* = 0.12) (9). Another thiotepa-containing regimen composed of thiotepa, etoposide, and melphalan was studied by a research group in Israel; they reported an improvement in PFS (44 months vs. 17 months), but with more adverse reactions in the study group (21). The previous three drug combinations appear to be more toxic than HD-MEL. Recently, double alkylator-based regimens have been proposed as a conditioning regimen in ASCT for MM. The BUMEL regimen is a representative conditioning regimen that has been shown to be effective in previous studies (22–25). Another regimen using thiotepa (275 mg/m^2^) and melphalan (140 mg/m^2^) in a second ASCT for MM patients was studied by an Italian group; it was found to be safe and effective with 2-year PFS and OS rates of 71.0% and 88.9%, respectively, and grade 3–4 mucositis incidence of only 9% (26). Reducing the dose of melphalan and adding busulfan or thiotepa is effective and does not appear to significantly increase the mucositis risk compared with HD-MEL. And unlike melphalan, busulfan and thiotepa do not require dose adjustment for decreased renal function. The combination of busulfan and thiotepa without melphalan was notably as effective as HD-MEL in this study.

Interestingly, the incidence of stomatitis and diarrhea was significantly lower in the BuTT group than in the HD-MEL group. Although there was no statistical difference, the occurrence of infection was also lower in the BuTT group, possibly due to the reduction of mucositis. The thiotepa-containing regimens used in the past comprised three consecutive days of 150–250 mg/m^2^/day thiotepa and oral busulfan (9–11). In this study, a relatively reduced dose of thiotepa (10 mg/kg) and intravenous busulfan were used; the regimen was tolerable for most patients. The renal function before ASCT was not evaluated in this study; however, no dose reduction was required for thiotepa when administered to patients with a reduced glomerular filtration rate. Additionally, it is of some concern that the simultaneous use of two alkylating agents could increase the VOD risk. However, there was no increase in the incidence of VOD, and no severe VOD was reported. Every patient who received BuTT was younger than 65; we were unable to analyze the outcome of older patients in this study. However, the BuTT conditioning regimen showed more prominent PFS improvement in a subgroup of patients older than 60 years; the reason is related to a favorable toxicity profile for BuTT. Therefore, the BuTT conditioning regimen is expected to be applied safely without dose reduction in older myeloma patients or patients with impaired renal function. Further research is necessary to define the efficacy and safety of BuTT conditioning in older patients.

This study had several limitations. The induction and maintenance treatments were different between the two groups; this is likely due to the retrospective nature of this research. There are some confounding factors in this study, such as a greater proportion of patients receiving bortezomib-containing induction treatment in the BuTT group, as well as a greater number of patients receiving maintenance treatment after transplantation in the HD-MEL group. We performed various subgroup analyses to minimize such confounding factors. However, further prospective study is needed to confirm the efficacy of BuTT conditioning. Additionally, minimal residual disease status and further response assessment more than 3 months after ASCT were not analyzed. Thus, the reason for the prolonged PFS, despite the similar ORR to BuTT, was not clarified in this study. However, similar findings have also been reported in a previous study of BUMEL, in which it has been suggested that busulfan changes the bone marrow microenvironment to an unfavorable state for myeloma cells besides having direct cancer cell toxicity (25, 27). Lastly, cytogenetic abnormalities were not analyzed in 7.9% of the patients, and the cut-off level defining del(17p) was inconsistent among the participating institutions. Therefore, it was difficult to evaluate the survival outcome according to the cytogenetic risk group. However, despite such limitations, the results of this study are meaningful in that they suggest the use of BuTT as another effective and safe conditioning regimen for MM patients.

The results of this study indicate consideration of the BuTT as an effective alternative conditioning regimen with reduced toxicity in patients with MM. Further randomized controlled prospective trials are required to confirm the efficacy of BuTT conditioning.

## Data availability statement

The raw data supporting the conclusions of this article will be made available by the authors, without undue reservation.

## Ethics statement

The studies involving human participants were reviewed and approved by the Institutional Review Board of Chonnam National University Hwasun Hospital. Written informed consent for participation was not required for this study in accordance with national legislation and institutional requirements.

## Author contributions

C-KM and J-JL designed the study; G-YS and S-HJ prepared the manuscript; and JK, HE, JM, H-YY, and KK critically reviewed the manuscript. All authors contributed to the article and approved the submitted version.

## Funding

This study received financial support from the Korea Otsuka: Research Funds for Study Management.

## Conflict of interest

The authors declare that the research was conducted in the absence of any commercial or financial relationships that could be construed as a potential conflict of interest.

## Publisher’s note

All claims expressed in this article are solely those of the authors and do not necessarily represent those of their affiliated organizations, or those of the publisher, the editors and the reviewers. Any product that may be evaluated in this article, or claim that may be made by its manufacturer, is not guaranteed or endorsed by the publisher.
